# Household Composition May Modify the Association Between Home Cooking and Dietary Diversity Among Japanese Corporate Employees

**DOI:** 10.3390/nu18111704

**Published:** 2026-05-27

**Authors:** Hitomi Matsuura, Eri Hiraiwa, Kanako Deguchi, Chihiro Ushiroda, Risako Yamamoto-Wada, Katsumi Iizuka

**Affiliations:** 1 Department of Clinical Nutrition, School of Medicine, Fujita Health University, Toyoake 470-1192, Japan; 51023103@fujita-hu.ac.jp (H.M.); 51022099@fujita-hu.ac.jp (E.H.); kanasakuran@gmail.com (K.D.); chihiro.ushiroda@fujita-hu.ac.jp (C.U.); risako.wada@fujita-hu.ac.jp (R.Y.-W.); 2 Faculty of Medicine, Fujita Health University, Toyoake 470-1192, Japan

**Keywords:** family composition, home cooking, dietary diversity, dietary structure, lifestyle context

## Abstract

**Background:** Dietary diversity, commonly assessed using the Shannon index, reflects dietary structure and food-group variety. However, whether dietary diversity is more closely associated with demographic characteristics or lifestyle-related factors remains unclear. **Objective:** To examine associations between dietary diversity based on 10 food groups and demographic and lifestyle-related factors among Japanese corporate employees. **Methods:** This cross-sectional study analyzed data from 925 company employees aged 20–59 years. The Shannon index was calculated from 10 food groups. Associations with age, sex, body mass index (BMI), workplace location, occupation, household composition, home cooking, and lunch type were evaluated using analysis of variance, analysis of covariance (ANCOVA), and permutational multivariate analysis of variance (PERMANOVA). **Results:** Dietary diversity was more closely associated with lifestyle-related factors, particularly household composition and home cooking, than with demographic characteristics. Individuals living alone exhibited lower Shannon index values than those living in multiperson households. Notably, home cooking was associated with greater dietary diversity among participants in multiperson households, whereas no such association was observed among individuals living alone. PERMANOVA analyses also suggested differences in dietary structure according to household composition. **Conclusions:** Household composition may influence dietary structure and may modify the association between home cooking and dietary diversity. These findings suggest that determinants of dietary diversity may be context-dependent.

## 1. Introduction

Dietary diversity is generally considered an important component of a balanced diet and is reflected in dietary guidelines recommending the consumption of a wide variety of foods across food groups to support nutritional adequacy [1,2]. In line with this concept, dietary diversity, often assessed using indices such as the Shannon index, has been associated with favorable health outcomes, including reduced mortality and improved nutritional status [3,4,5,6]. Because dietary patterns may capture overall eating behavior more effectively than individual nutrients, diversity-based indices provide a practical approach to evaluating dietary structure and food-group variety [7,8,9,10,11]. However, dietary diversity does not necessarily indicate healthier eating behavior, and some studies have suggested that diversity indices may also reflect variation derived from processed or nutritionally imbalanced foods [12]. For example, dietary diversity indices may also capture diversity derived from ultra-processed or nutritionally imbalanced foods and therefore do not necessarily reflect overall diet quality [12,13,14]. Recent studies have highlighted potential health risks associated with ultra-processed foods, including artificial sweeteners, emulsifiers, and industrial additives [12,13]. In addition, associations between dietary diversity and health outcomes have not always been consistent across studies [14].

Previous studies, including our own, have reported associations between dietary diversity and demographic characteristics such as age and sex [15]. However, these findings have often been derived from relatively homogeneous populations, such as healthcare professionals, in whom variability in lifestyle factors may be limited. As a result, the extent to which dietary diversity is shaped by individual attributes versus daily living conditions remains unclear.

Lifestyle-related factors, including household composition and home cooking, may directly influence food selection and meal patterns and may better reflect the social and behavioral context of eating than demographic characteristics alone [16,17,18]. However, their relative importance in determining dietary diversity has not been fully examined.

Therefore, the present study aimed to determine whether dietary diversity is more strongly influenced by lifestyle-related factors than by demographic characteristics among corporate employees. Specifically, we examined the effects of workplace region, occupation, household size, home cooking, and lunch style, in addition to age, sex, and BMI. By clarifying the relative importance of these factors, this study may contribute to understanding the context-dependent determinants of dietary diversity and dietary structure.

## 2. Materials and Methods

### 2.1. Study Design and Participants

This retrospective cross-sectional observational study aimed to clarify the differences in eating patterns with respect to family composition and home cooking. From 26 January to 6 February 2026, a questionnaire survey, including a food frequency questionnaire, was conducted for employees of Rohto Pharmaceutical, and data from 987 participants were provided to us in a deidentified and anonymized format. Responses from individuals who provided their sex and were between 20 and 59 years old were selected, resulting in the use of data from 925 individuals (220 men and 705 women). The food frequency questionnaire was provided in a fully anonymized form, and thus the data could not be linked to any specific individual. In this study, we analyzed data that were already anonymized, making the identification of individuals impossible. Therefore, we have announced on our website of the Department of Clinical Nutrition, Fujita Medical School (HM25-323, 5 November 2025; HM25-704, 14 April 2026) that exclusion was not possible because individuals cannot be identified from de-identified and anonymized data. This study was conducted in accordance with the principles of the Declaration of Helsinki and approved by the Research Ethics Committee of Fujita Health University (HM25-323, 5 November 2025; HM25-704, 14 April 2026).

### 2.2. Questionnaire on Location, Job Type, Home Cooking, Dining Area, Family Composition, and Food Groups

The answers to the questions were collected and compiled using Google Forms. Responses were gathered anonymously. The questions included height, weight, workplace location (Tokyo, Osaka, Mie (Ueno), Kyoto (RVK), or other locations), job type (production/technical, administrative, R&D, sales, or other jobs), home cooking (yes/no), dining environment (convenience store, eating out, employee cafeteria, healthy lunch, homemade lunch, or none), and family size (1 person, 2 persons, 3 persons, 4 persons, or 5+ persons).

In the present study, demographic variables included age group, sex, BMI, and workplace location, whereas lifestyle-related variables included household composition (family size), home cooking, and dining environment. Demographic variables were included as covariates in the multivariable models when evaluating associations between lifestyle-related variables and dietary diversity.

Furthermore, participants were asked about the frequency of consumption (number of times per week) of meat, fish, eggs, dairy products, soy products, green and yellow vegetables, seaweed, fruits, and potatoes, and intake frequency per day was calculated [12]. Fats and oils (butter or margarine) were treated as regularly consumed food items because these ingredients are commonly incorporated into routine cooking and meal preparation in the Japanese diet.

Dietary diversity was evaluated using the Shannon diversity index based on 10 food groups (meat, fish, eggs, milk, soy products, vegetables, fruits, seaweed, potatoes, and fats/oils). The 10 food groups used in the present study were based on food-group classifications commonly used in Japanese dietary diversity assessments, particularly in studies involving older adults and community-based nutritional assessment [19]. These food groups were selected because they broadly capture major sources of protein, dietary fiber, and micronutrients in the Japanese diet.

For each participant, intake values for each food group were converted into proportions relative to the total intake across all food groups. The Shannon index was then calculated as:

H′ = −∑ pi ln(pi)

where pi represents the proportion of weekly intake frequency for each food group, and S represents the total number of food groups. Higher values indicate greater dietary diversity. However, some dietary components, such as staple foods, beverages, and ultra-processed foods, were not included in the diversity calculation. Therefore, in the present study, the Shannon index was interpreted primarily as a measure of food-group diversity and dietary structure rather than as a comprehensive assessment of total diet quality.

### 2.3. Statistical Analysis

#### 2.3.1. Associations Between Household and Lifestyle Factors and Dietary Diversity (Shannon Index)

Dietary diversity was quantified using the Shannon index, which was calculated from individual-level food group intake data. We performed a Type III analysis of variance (ANOVA) within a general linear modeling framework (N = 925) to examine the associations between household and lifestyle factors and dietary diversity. Type III sums of squares were used to evaluate the independent contribution of each factor after adjustment for the other variables in models including interaction terms and unequal subgroup sizes. The dependent variable was the Shannon index. The independent variables included age group (4 categories), sex (male/female), age group × sex interaction, family size (5 categories), location (5 categories), home cooking frequency (2 categories), dining area (6 categories), job type (5 categories), and body mass index (BMI; continuous). All categorical variables were entered as fixed factors. BMI was included as a continuous covariate. Type III sums of squares were used to estimate the main effects while adjusting for all other variables in the model. The results obtained from the model are reported as F statistics with corresponding degrees of freedom, p values, partial eta squared (η^2^p) values as a measure of the effect size, and 95% confidence intervals (CIs) for η^2^p. Statistical significance was defined as a two-sided *p* value < 0.05. Analyses were conducted using R (R Foundation for Statistical Computing, Vienna, Austria) [20]. The Type III ANOVA was performed using the car package.

#### 2.3.2. Associations Between Family Size and the Shannon Diversity Index of Food Intake

Next, we compared whether the Shannon index varied based on the family structure. Specifically, we calculated the Shannon diversity index from food intake frequency and constructed a linear model using family size (five groups: 1, 2, 3, 4, and 5 or more people) as the main explanatory variable. We tested the effects of each factor using Type III ANOVA. By targeting individuals in their 20s to 50s, we adjusted for covariates such as BMI, occupation, and region. We also constructed an interaction model (age group × sex), compared the AIC values, calculated the effect size (η^2^), and conducted Tukey’s post hoc tests.

#### 2.3.3. Redundancy Analysis (RDA)

On the basis of the Shannon index, dietary diversity differed between individuals living alone and those living with two or more people. Redundancy Analysis (RDA) was conducted to identify which food components distinguished the two groups. The household composition was categorized into single-person (single) and multiperson (multiperson) households based on the self-reported family size. Intakes of 10 food groups (meat, fish, eggs, milk, soy products, vegetables, fruits, seaweed, potatoes, and fats/oils) were used to calculate Bray–Curtis dissimilarities between individuals, and a permutational multivariate analysis of variance (PERMANOVA) was performed with the household composition as the grouping factor; the homogeneity of multivariate dispersions was evaluated using betadisper. Using the same distance matrix and grouping factor, we then conducted distance-based redundancy analysis (db-RDA; capscale function in the vegan package) and visualized the distribution of sample scores on the first constrained axis (CAP1) and the corresponding food loadings.

#### 2.3.4. Dietary Intake Frequency by Household Type: Adjusted Comparisons Across Food Categories (ANCOVA)

We conducted analysis of covariance (ANCOVA) using two models to quantify the contributions of individual food groups suggested by the db RDA. In both models, the intake frequency of each food group (meat, fish, eggs, milk, soy products, vegetables, fruits, seaweed, potatoes, and fats/oils) was treated as the dependent variable, and the household composition (single-person vs. multi-person households) was treated as the main factor. In Model 2, we adjusted for age group and sex; fit linear models of the form “food intake ~ household composition + age group + sex”; and obtained F values, partial eta squared values, and Benjamini–Hochberg adjusted P values for the effect of the household composition. In Model 3, BMI was additionally included as a covariate (“food intake ~ household composition + age group + sex + BMI”) to assess whether the associations were robust to further adjustment for adiposity and to perform a sensitivity analysis.

#### 2.3.5. Interactive Effects of the Household Type and Homemade Cooking on Dietary Diversity: Type III ANOVA

We analyzed participants younger than 60 years and calculated a Shannon diversity index for dietary intake based on the frequency of consumption of ten food groups (meat, fish, eggs, milk, soy, vegetables, fruits, seaweed, potatoes, and oils). Using this index as the dependent variable, we fitted multiple linear regression models including the living arrangement (single-person vs. multiperson households) and home cooking frequency as the main predictors and age group, sex, residential area, eating out location, job type, and BMI as covariates. Type III analysis of variance was performed for a main effects model and for an interaction model including the living arrangement × home cooking interaction term. When the interaction was statistically significant, we conducted simple effect analyses using estimated marginal means with Tukey-adjusted pairwise comparisons to examine differences in Shannon diversity across living arrangements and home cooking frequency levels.

## 3. Results

### 3.1. Baseline Demographic, Dietary, and Lifestyle Characteristics by Household Type

In terms of the participants’ background, 925 study participants were aged 20–59 years (44% male and 56% female). Among single-person households (220 people), the largest age group was participants in their 20s, with 68 individuals (30.9%) (Table 1). Conversely, among households with two or more people (705 individuals), the smallest group was those in their 20s, with 54 people (7.7%), while the largest group was those in their 40s, with 272 people (38.6%). BMI was lower in single-person households (21.97 ± 3.26) than in households with two or more people (22.55 ± 3.57). In terms of the place of work, 339 workplaces were in Osaka (36.6%), 103 were in Kyoto (11.1%), 118 were in Tokyo (12.8%), 277 were in Mie (Ueno) (29.9%), and 88 were in other locations (9.5%). With respect to occupation, among those living alone, the most common occupations were production/technical (76 people; 34.5%), followed by R&D (63 people; 28.6%). Among participants living with two or more people, the most common occupations were production/technical at 290 (41.1%), followed by administrative at 193 (27.4%). The number of people who did not cook for themselves was greater among those living alone (32 people, 14.5%) than among those living with two or more people (10 people, 1.4%). With respect to lunchtime meals, 109 people (11.8%) purchased meals from convenience stores, 136 (14.7%) ate at restaurants, 551 (59.6%) used the employee cafeteria, 3 (0.3%) ordered the delivery of healthy lunchboxes, 99 (10.7%) brought homemade lunchboxes, and 27 (2.9%) did not have lunch. In terms of household composition, 220 single-person households (23.8%), 204 two-person households (22.1%), 208 three-person households (22.5%), 207 four-person households (22.4%), and 86 households with five or more people (9.3%) were included (Table 1).

### 3.2. Associations Between Household and Lifestyle Factors and the Dietary Diversity (Shannon Index): Type III ANOVA

In our previous report, the Shannon index was associated with age and sex. Therefore, in this study, we first decided to examine the main effects of the age group (four groups) and sex (two groups), as well as the interaction between the age group × sex to investigate “differences by age and sex.” Furthermore, covariates (adjustment variables), including family size, location, home cooking frequency, dining area, job type (all categorical), and BMI (continuous), were included together in the model to control for their effects, allowing us to evaluate the effects of age, sex, and their interaction. After adjusting for the other factors, the results revealed that the family composition (η^2^p = 0.027 [0.010, 0.047]) and home cooking frequency (η^2^p = 0.007 [0.001, 0.021]) were more strongly associated with the Shannon index than the age group × sex interaction was (η^2^p = 0.001 [0.000, 0.005]) (Table 2). Therefore, in the following section, we focus on the family composition and home cooking frequency and examine their detailed association patterns.

### 3.3. Relationships Between the Shannon Index and Family Structure

Next, we compared whether the Shannon index varied based on the family structure. 

**Figure 1:**
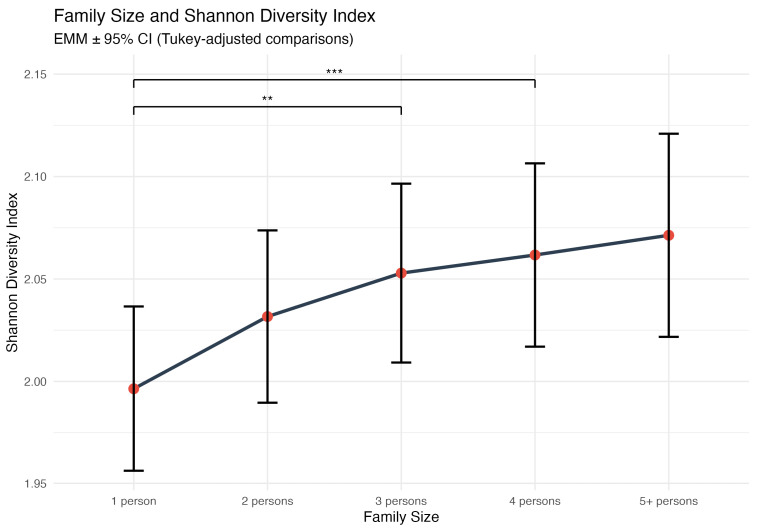
Estimated marginal means (EMMs) of the Shannon dietary diversity index by family size. The Shannon index was calculated using self-reported intake frequencies of 10 food groups (meat, fish, egg, milk, soy, vegetables, fruits, seaweed, potatoes, and oil). The x-axis represents family size (1, 2, 3, 4, and 5+ persons), and the y-axis represents adjusted EMMs of the Shannon diversity index. Error bars indicate 95% confidence intervals. EMMs were estimated using ANCOVA with Type III ANOVA. Significant pairwise differences identified by Tukey’s post hoc test are indicated by brackets (** *p* < 0.01, *** *p* < 0.001).

Family size was significantly positively associated with the Shannon diversity index (F = 6.45, *p* < 0.001; partial η^2^ = 0.028 [small-to-moderate effect size]). In the subsequent Tukey test, single-person households were significantly different from households with 3, 4, or 5+ people. The estimated marginal means (95% confidence intervals) were as follows: 1 person—1.973 (1.922–2.025), 2 persons—2.009 (1.956–2.062), 3 persons—2.030 (1.976–2.084), 4 persons—2.039 (1.983–2.094), and 5+ persons—2.050 (1.992–2.109) (Table 3, Figure 1).

Among the covariates, whether the person cooked for themselves (self-cooking) also showed a significant association with the Shannon diversity index (F = 5.82, *p* = 0.016, partial η^2^ = 0.006). Compared with the model that only included the main effects, the model including the age × sex interaction resulted in a slightly greater AIC (−978.5 vs. −983.4) and showed no improvement in fit; thus, the main effects model was used for the interpretation.

In summary, these results indicate that family structure is positively associated with the Shannon index, a measure of dietary diversity.

### 3.4. RDA Analysis

On the basis of the Shannon index, dietary diversity differed between individuals living alone and those living with two or more people. RDA was conducted to identify which food components distinguished the two groups. Specifically, Bray–Curtis distances were calculated from the intake frequency of 10 food items. First, PERMANOVA (adonis2) was used to test differences in dietary patterns according to the household composition, and, concurrently, betadisper was used to confirm the homogeneity of variances between groups. Furthermore, using the same distance matrix and group variables, a distance-based redundancy analysis (db-RDA; capscale) was performed to visualize the distribution of sample scores on the first constrained axis (CAP1) and the food loadings.

**Figure 2:**
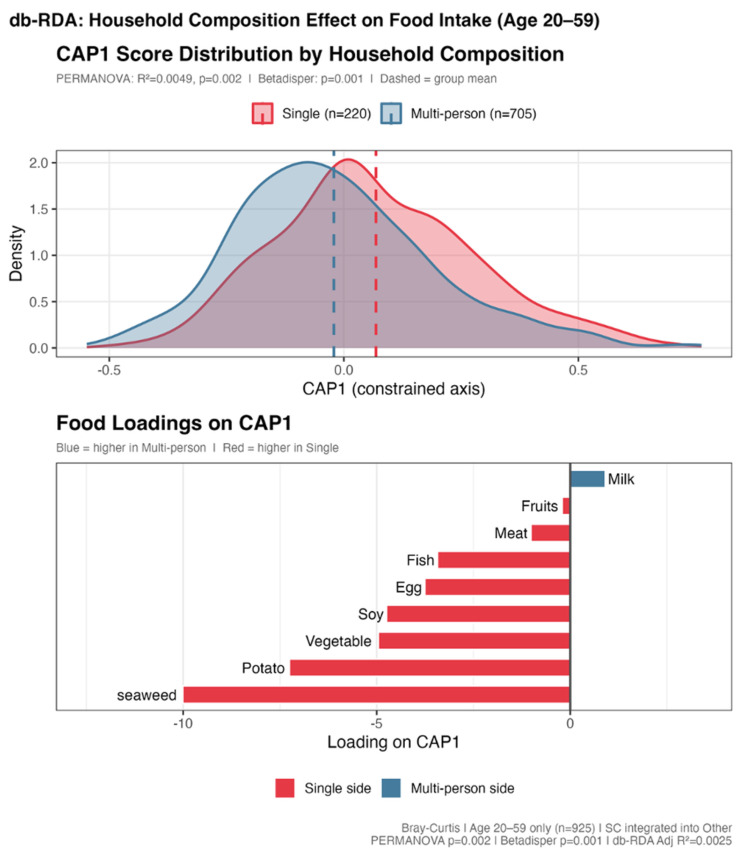
Distance-based redundancy analysis (db-RDA) of food intake patterns by household composition (aged 20–59 years).

PERMANOVA (adonis2) using the Bray–Curtis distance revealed a significant difference in food intake patterns between single-person households and multiperson households (R^2^ = 0.0049, *p* = 0.002). In addition, the test of multivariate dispersion based on betadisper (permdisp) was also significant (F = 13.17, *p* = 0.001), suggesting that food patterns in single-person households are more varied than those in multiperson households are. Moreover, the results of the RDA and individual variance analyses for each food item indicated that seaweed, fish, potatoes, and eggs were consumed in significantly greater amounts in multiperson households, and their effect sizes were greater than those for other foods (seaweed: η^2^ = 0.020, ω^2^ = 0.019, F = 19.09, p_FDR < 0.001; potato: η^2^ = 0.017, ω^2^ = 0.016, F = 16.23, p_FDR < 0.001; fish: η^2^ = 0.016, ω^2^ = 0.015, F = 14.62, p_FDR < 0.001; and eggs: η^2^ = 0.0087, ω^2^ = 0.0076, p_FDR = 0.010). On the other hand, for the other foods, no significant differences were observed between single-person and multiperson households (Figure 2).

A Bray–Curtis dissimilarity matrix was constructed from self-reported intake frequencies of 10 food groups (meat, fish, eggs, milk, soy products, vegetables, fruits, seaweed, potatoes, and fats/oils) and analyzed using distance-based redundancy analysis (db-RDA). (A) Distribution of CAP1 scores according to household type. Red and blue curves indicate single-person and multiperson households, respectively; dashed vertical lines denote group means. PERMANOVA R^2^ and *p* values, together with betadisper *p* values, are shown in the subtitle. (B) Food-group loadings on CAP1. Positive loadings indicate food groups more strongly associated with multiperson households, whereas negative loadings indicate food groups more strongly associated with single-person households. Multivariate differences in dietary structure were evaluated using PERMANOVA based on Bray–Curtis dissimilarity, and homogeneity of multivariate dispersion was assessed using betadisper.

Therefore, although differences in variance between groups were observed with respect to permdisp, the results from PERMANOVA also revealed that the group centroids differed; in particular, the intake of seaweed, fish, potatoes, and eggs was considered to contribute to the differences based on household size.

### 3.5. Dietary Intake Frequency by Household Type: Adjusted Comparisons Across Food Categories (ANCOVA)

**Figure 3:**
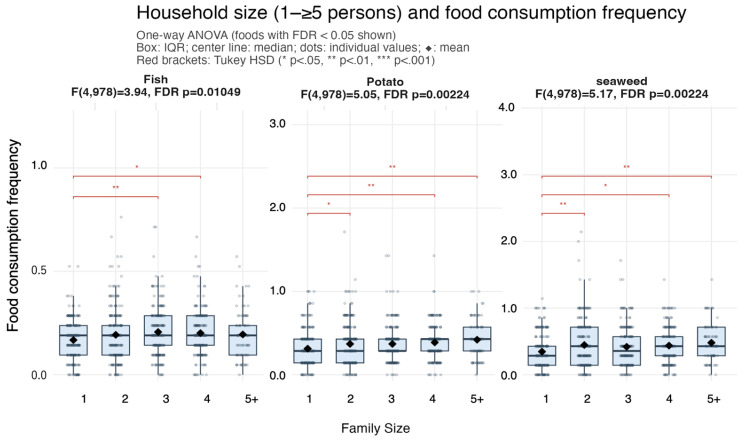
Distribution of food intake frequency by household size (one-way ANOVA; FDR < 0.05 only). Box-and-jitter plots are shown for food groups with a Benjamini–Hochberg false discovery rate (FDR)-adjusted *p* value < 0.05 in one-way ANOVA across household-size groups (1, 2, 3, 4, and ≥5 persons). Boxes indicate interquartile ranges, center lines indicate medians, and whiskers extend to 1.5 × the interquartile range. Gray dots represent individual observations, and black dots represent group means. Significant pairwise comparisons were assessed using the Tukey–Kramer method.

Furthermore, ANCOVA was conducted using two models to quantitatively evaluate the degree of contribution of each food item identified by the db-RDA. In Model 2, a covariance analysis was performed with the intake frequency of each food as the dependent variable, household composition (single-person vs. multiperson) as the main effect, and age group and sex as covariates. This approach allowed for the assessment of intake differences by household composition adjusted for age and sex. In Model 3, BMI was added as an additional covariate, and ANCOVA was conducted to confirm whether the results remained consistent after adjustment for BMI. In Model 2, the intake frequencies of fish, eggs, seaweed, and potatoes were significantly lower for single-person households than for multiperson households (fish: Diff = −0.0243, F = 7.674, partial η^2^ = 0.0078, p_FDR = 0.013; egg: Diff = −0.0380, F = 8.606, partial η^2^ = 0.0087, p_FDR = 0.010; seaweed: Diff = −0.0754, F = 10.737, partial η^2^ = 0.0109, p_FDR = 0.005; potato: Diff = −0.0692, F = 15.355, partial η^2^ = 0.0155, p_FDR < 0.001) (Table 4, Figure 3). A similar trend was also observed in Model 3, in which BMI was further adjusted. Therefore, in accordance with the db-RDA findings, these four food items (especially potatoes and seaweed) were suggested to contribute primarily to the differences in dietary patterns between single-person and multiperson households (Table 4, Figure 3).

### 3.6. Post Hoc Comparison of Dietary Diversity (Shannon Index) by Household Type and Homemade Cooking: Estimated Marginal Means and Simple Main Effects

**Figure 4:**
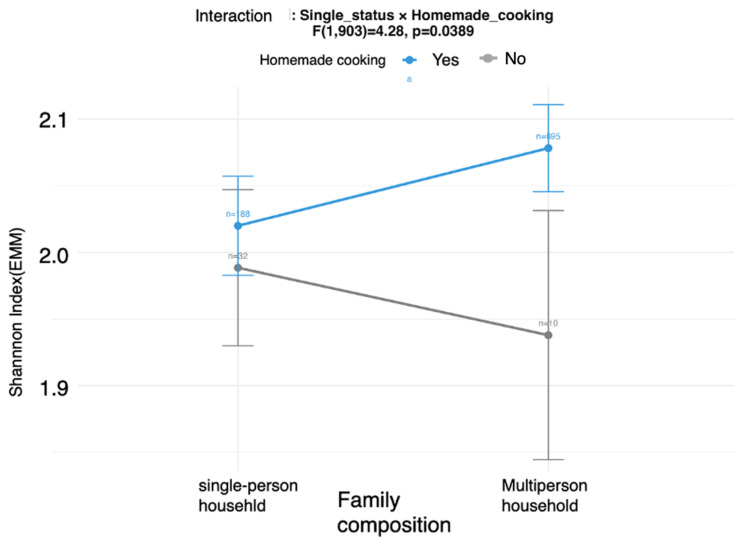
Effect of the interaction between household composition and home-cooking status on the Shannon index of dietary diversity (Type III). The x-axis represents the household composition (single-person vs. multiperson households), and the y-axis represents the estimated marginal mean (EMM) of the Shannon dietary diversity index. The Shannon index was calculated as −Σ(pᵢ log pᵢ) from the proportional intake frequencies of 10 food groups (meat, fish, egg, milk, soy, vegetables, fruits, seaweed, potatoes, and oil). Lines are colored according to the home cooking status: blue (yes) indicates regular home cooking, and gray (no) indicates no regular home cooking. The error bars represent 95% confidence intervals (CIs). Sample sizes (n) per cell are displayed above each point. The F statistic and p value for the single status × home cooking interaction term from a linear model with Type III sums of squares (car::ANOVA), adjusted for age group (categorical), sex, residential area, dining location, job type, and BMI, are shown in the figure title. EMMs were estimated using the emmeans package. This figure is presented only when the interaction term was statistically significant (*p* < 0.05). The analyses were restricted to participants aged 20–59 years; individuals aged ≥60 years were excluded, and the occupational category “SC” was merged into “Other”. Subgroup sample sizes were: Single/No cooking (n = 32), Single/Yes cooking (n = 188), Multiperson/No cooking (n = 10), and Multiperson/Yes cooking (n = 695).

As described above, household composition was associated with differences in dietary structure, suggesting that living arrangements may shape daily eating environments and meal patterns. Therefore, we further examined whether the association between home cooking and dietary diversity differed according to household composition using a Type III analysis of variance including an interaction term between household composition and home cooking. Multiple regression model was constructed with the Shannon diversity index as the dependent variable and, in addition to living alone or with others and cooking frequency, the age group, sex, residential area, dining out location, occupation, and BMI were used as independent variables. A Type III analysis of variance was performed on both the main effects model and the interaction model (living alone × home cooking), and the interaction term between cooking frequency and family composition was significant [F(4, 903) = 4.20, *p* = 0.039] (Table 5, Figure 4). A post hoc analysis of simple effects was conducted using emmeans to examine the nature of the interaction. In comparisons regarding the presence or absence of cooking, the difference between “no home cooking vs. home cooking” among those living alone was not significant [estimate = −0.0315, SE = 0.0271, *p* = 0.245] (Table 5, Figure 4). On the other hand, in multiperson households, the Shannon index was significantly higher for those who cooked at home than for those who did not [estimate = −0.1402, SE = 0.0451, *p* = 0.002], indicating that home cooking was associated with increased dietary diversity. With respect to the effect of the family composition, a significant difference in the Shannon index was not observed between single-person and multiperson households when the participants did not cook at home [estimate = 0.0506, SE = 0.0514, *p* = 0.325], but among those who cooked at home, the Shannon index was significantly higher in multiperson households [estimate = −0.0582, SE = 0.0123, *p* < 0.001] (Table 5, Figure 4). In summary, when home cooking is the dependent variable, living in a multiperson household is associated with greater dietary diversity than living alone. Conversely, while cooking at home increases dietary diversity in multiperson households, no such difference was observed for those living alone, regardless of whether they cooked.

Although the observed interaction effect size was modest, these findings suggest that differences in household composition may shape dietary structure and meal environments, thereby modifying the effect of home cooking on dietary diversity. In this context, home cooking may not uniformly promote dietary diversity, but rather its association may depend on household-related eating environments and daily meal structure.

## 4. Discussion

In this study, we examined the relative contributions of demographic characteristics and lifestyle factors to dietary diversity, assessed using the Shannon index. We found that dietary diversity was more strongly associated with lifestyle factors, particularly household composition and home cooking, than with demographic characteristics such as age and sex. Individuals living in multiperson households exhibited greater dietary diversity than those living alone. Notably, the effect of home cooking differed by household type: in multiperson households, home cooking was associated with higher dietary diversity, whereas no such association was observed among individuals living alone. These findings suggest that lifestyle-related factors may be more strongly associated with dietary diversity than demographic characteristics in this occupational cohort. Furthermore, these findings suggest that the determinants of dietary diversity may be context-dependent and that household-related lifestyle context, particularly household composition, may be important in shaping dietary diversity patterns.

The Shannon index was used in this study to assess dietary diversity, as it captures both the richness and evenness of food intake and has been validated as an indicator of food biodiversity [21]. Previous studies have demonstrated that the Shannon index is positively correlated with the intake of vegetables, fruits, dietary fiber, and key micronutrients [22]. In the present study, dietary diversity was evaluated based on the balanced consumption of ten food groups, supporting the utility of the Shannon index as a practical measure of overall dietary patterns. However, dietary diversity does not necessarily indicate healthier eating behavior. Diversity indices may also capture variation derived from processed or nutritionally imbalanced foods and, therefore, should be interpreted as indicators of dietary structure and food variety rather than direct measures of overall diet quality. Therefore, in the present study, the Shannon index was primarily used to evaluate dietary structure and food-group variety rather than to directly assess the healthfulness of the diet.

The PERMANOVA analysis demonstrated statistically significant differences in dietary structure according to household composition; however, the observed R^2^ values were relatively small. This suggests that household composition explains only a limited proportion of the variability in dietary structure and that dietary behaviors are likely influenced by multiple additional factors, including socioeconomic, occupational, and food-environment-related variables. Because dietary behaviors are complex and multifactorial, household composition would not be expected to explain a large proportion of total variability in dietary structure. Furthermore, the significant betadisper results suggest heterogeneity of dispersion between groups, indicating that the observed differences may reflect not only differences in group centroids but also variability in dietary patterns within groups.

In the ANCOVA analyses of individual food-group intake frequencies, several effect sizes were relatively modest despite statistical significance. Because these variables were based on self-reported food-frequency measures reflecting habitual dietary behaviors rather than clinical or physiological outcomes, large effect sizes may be less likely to occur. Therefore, the observed associations should be interpreted primarily in terms of differences in dietary structure and eating environments at the population level rather than individual-level clinical effects.

Our findings are consistent with previous studies demonstrating that shared meals and home cooking are associated with improved diet quality and increased intake of diverse food groups. For example, the Project EAT study reported that a higher frequency of shared meals was associated with increased consumption of fruits, vegetables, and essential nutrients [23]. Similarly, the Fenland Study showed that frequent home cooking was associated with higher vegetable and fruit intake and better diet quality scores [24]. These findings support the notion that lifestyle factors contribute to dietary diversity.

Several mechanisms may explain these associations. Household composition influences meal structure, food sharing, and the number of dishes prepared, thereby increasing dietary variety. In multiperson households, preparing meals for several individuals may encourage the use of a wider range of ingredients. In addition, social facilitation during shared meals has been shown to increase both food intake and dietary variety; for example, individuals consume more food when eating with others than when eating alone [16], and this effect is consistently observed across studies [17]. Furthermore, higher-quality family meals have been associated with improved dietary quality and psychosocial health [25]. Together, these findings suggest that lifestyle factors shape dietary diversity through the social context of eating.

Importantly, individuals living alone exhibited lower dietary diversity regardless of whether they cooked for themselves. This finding suggests that the benefits of home cooking may depend on household context. One possible explanation is the lack of economies of scale in single-person households, where preparing a variety of dishes may be less practical and economical. Consistent with this interpretation, an analysis of KNHANES data reported that dietary quality scores were lower among individuals living alone, partly due to greater reliance on eating out and processed foods [26]. These findings highlight structural differences in dietary behavior between single- and multiperson households.

Interestingly, home cooking was not associated with greater dietary diversity among individuals living alone. One possible explanation is that single-person households may experience practical constraints such as difficulty purchasing diverse ingredients in small quantities, concerns regarding food waste, and the burden of preparing multiple dishes for one person. In modern urban environments, “home cooking” may also include simplified meal preparation using ready-made foods, pre-cut ingredients, or convenience-oriented meal assembly rather than cooking from raw ingredients [27]. These factors may contribute to simpler and more repetitive dietary structures, potentially limiting dietary diversity even among individuals who prepare meals at home.

Taken together, these results suggest that dietary diversity may be more closely associated with lifestyle-related factors than demographic characteristics in this occupational cohort. While age and sex may capture broad population differences, lifestyle factors such as household composition and home cooking may more directly reflect daily eating environments and dietary behaviors. This may partly explain why demographic associations observed in previous studies were less evident in the present study. Moreover, because subgroup sizes were highly unbalanced, particularly in the Single × No home cooking subgroup (n = 10), elevated multicollinearity was observed in the interaction model (generalized VIF ≈ 5), and the interaction results should therefore be interpreted cautiously. Therefore, the interaction results should be interpreted cautiously. These observations may further support the concept that the determinants of dietary diversity are context-dependent.

In our previous study, we observed a significant interaction between age and sex in relation to dietary diversity [15]. In the present study, we also included age, sex, and their interaction term in the analysis; however, neither the main effects nor the interaction was significantly associated with the Shannon index. This discrepancy likely reflects differences in population structure rather than inconsistency in the underlying relationships. In the previous study, which focused on healthcare professionals, lifestyle-related variables such as household composition and home cooking were not included, and variability in these factors may have been limited [15]. In contrast, the present study explicitly incorporated these lifestyle factors within a more heterogeneous population with respect to living arrangements and daily routines. When included in the model, household size and home cooking emerged as significant determinants of dietary diversity, whereas demographic characteristics did not. These findings suggest that associations previously attributed to demographic characteristics may, at least in part, be explained by underlying lifestyle context. This interpretation is consistent with demographic trends in Japan, including delayed marriage and the increasing prevalence of single-person households, which may reduce opportunities for shared meals and diverse food consumption. Taken together, these results underscore the importance of considering population structure and lifestyle context when examining the determinants of dietary diversity.

This study has several limitations. First, its cross-sectional design precludes causal inference. Second, the study population was limited to employees of a single company, and workplace-specific factors such as work schedules, employee benefits, and the availability of company cafeterias may have influenced dietary behavior. In addition, because the cohort consisted of actively employed individuals, healthy worker bias cannot be excluded. The sex distribution was also imbalanced, which may limit the generalizability of the findings. However, the relatively homogeneous occupational environment may also have reduced variability related to workplace conditions. Therefore, caution is warranted when generalizing these findings to other populations, particularly those with different occupational or socioeconomic backgrounds. Furthermore, because the present study was exploratory and hypothesis-generating in nature, the findings should be interpreted cautiously and require validation in larger and more diverse populations.

Third, lifestyle-related variables were assessed in a simplified manner. In particular, home cooking was evaluated as a binary variable, and detailed aspects such as cooking frequency, meal complexity, and cooking motivation were not assessed. In addition, some subgroups—particularly participants in multiperson households who did not cook for themselves—were relatively small, which may have reduced the stability of subgroup estimates. These limitations may be particularly relevant when interpreting the context-dependent associations observed between household composition and home cooking. Future studies should evaluate cooking frequency, meal preparation complexity, and the use of convenience or ready-made foods in greater detail.

In addition, the Shannon index used in the present study was designed to evaluate food-group diversity and dietary structure rather than overall diet quality. Some dietary components, such as staple foods, beverages, and ultra-processed foods, were not included in the diversity calculation. Furthermore, fats and oils were treated as regularly consumed food items because these ingredients are commonly incorporated into routine cooking practices in the Japanese diet. However, this assumption may have reduced interindividual variability in this food category and may have influenced the Shannon diversity index calculation.

Fourth, we evaluated demographic factors (age group, sex, BMI, and workplace location) and lifestyle-related factors (household composition, home cooking, and dining environment) associated with dietary diversity. However, socioeconomic factors such as income and educational level were not assessed because of data protection constraints. These factors are likely to influence food choice, dietary diversity, and lifestyle-related behaviors; therefore, residual confounding related to socioeconomic and occupational factors cannot be excluded. However, because all participants were employees of a single company, socioeconomic variability may have been smaller than that in general population-based studies.

Finally, all data were based on self-reported questionnaires and were therefore subject to recall bias and social desirability bias. In addition, because participation was voluntary and anonymous, selection bias toward more health-conscious individuals cannot be ruled out, and the characteristics of nonrespondents could not be evaluated.

Future studies should incorporate more detailed assessments of lifestyle and socioeconomic factors, including food accessibility, work-related factors, the frequency of home cooking, and employ longitudinal and multi-institutional designs to validate and extend these findings in more diverse populations.

## 5. Conclusions

In conclusion, dietary diversity in this occupational cohort appeared to be more closely associated with lifestyle-related factors, particularly household composition and home cooking, than with demographic characteristics. Individuals living alone tended to exhibit lower dietary diversity regardless of whether they cooked for themselves, suggesting that household-related eating environments may influence dietary structure and food variety. In contrast, among participants living in multiperson households, home cooking was associated with greater dietary diversity. These findings support the concept that the determinants of dietary diversity may be context-dependent and influenced by household-related lifestyle environments. Consideration of household composition and daily meal environments may therefore be important when interpreting dietary diversity patterns and designing future dietary support strategies.

## Figures and Tables

**Table 1:** Baseline characteristics of participants by household type.

Variable	Overall (n = 925)	Single (n = 220)	Multiple (n = 705)	*p* Value	Statistical Test
N	**925**	220	705		
Sex, n (%) [Male]	407 (44.0%)	91 (41.4%)	316 (44.8%)		
**Age group, n (%)**				<0.001 ***	Chi-square
20s	122 (13.2%)	68 (30.9%)	54 (7.7%)		
30s	257 (27.8%)	60 (27.3%)	197 (27.9%)		
40s	317 (34.3%)	45 (20.5%)	272 (38.6%)		
50s	229 (24.8%)	47 (21.4%)	182 (25.8%)		
**BMI, mean ± SD**	22.41 ± 3.51	21.97 ± 3.26	22.55 ± 3.57	0.024 *	Mann–Whitney
Median [IQR]	21.78 [20.03–24.01]	21.28 [19.83–23.68]	21.91 [20.08–24.06]		
**Location, n (%)**				<0.001 ***	Chi-square
Osaka	339 (36.6%)	77 (35.0%)	262 (37.2%)		
Kyoto	103 (11.1%)	33 (15.0%)	70 (9.9%)		
Tokyo	118 (12.8%)	24 (10.9%)	94 (13.3%)		
Mie (Ueno)	277 (29.9%)	52 (23.6%)	225 (31.9%)		
Other	88 (9.5%)	34 (15.5%)	54 (7.7%)		
**Job type, n (%)**				0.001 **	Chi-square
Administrative	236 (25.5%)	43 (19.5%)	193 (27.4%)		
Production/Technical	366 (39.6%)	76 (34.5%)	290 (41.1%)		
R&D	189 (20.4%)	63 (28.6%)	126 (17.9%)		
Sales	116 (12.5%)	35 (15.9%)	81 (11.5%)		
Other	18 (1.9%)	3 (1.4%)	15 (2.1%)		
**Homemade cooking, n (%)**				<0.001 ***	Chi-square
No	42 (4.5%)	32 (14.5%)	10 (1.4%)		
Yes	883 (95.5%)	188 (85.5%)	695 (98.6%)		
**Dining area, n (%)**				0.498	Chi-square
Convenience store	109 (11.8%)	23 (10.5%)	86 (12.2%)		
Eat out	136 (14.7%)	37 (16.8%)	99 (14.0%)		
Employee cafeteria	551 (59.6%)	132 (60.0%)	419 (59.4%)		
Healthy lunch	3 (0.3%)	0 (0.0%)	3 (0.4%)		
Homemade lunchbox	99 (10.7%)	19 (8.6%)	80 (11.3%)		
None	27 (2.9%)	9 (4.1%)	18 (2.6%)		
**Family composition, n (%)**				<0.001 ***	Chi-square
1 person	220 (23.8%)	220 (100.0%)	0 (0.0%)		
2 persons	204 (22.1%)	0 (0.0%)	204 (28.9%)		
3 persons	208 (22.5%)	0 (0.0%)	208 (29.5%)		
4 persons	207 (22.4%)	0 (0.0%)	207 (29.4%)		
≥5 persons	86 (9.3%)	0 (0.0%)	86 (12.2%)		

IQR, interquartile range; R&D, research and development. * *p* < 0.05; ** *p* < 0.01; *** *p* < 0.001. *p* values were calculated using Chi-square test for categorical variables and Mann–Whitney U test for continuous variables.

**Table 2:** Associations Between Household and Lifestyle Factors and Dietary Diversity (Shannon Index): Type III ANOVA (N = 925).

Source	Df	MS	F(df_1_, df_2_)	P	η^2^p	95% CI	Sig.
Age group	3	0.006	*F(3, 898) = 0.32*	0.814	0.001	[0.000, 0.005]	
Sex	1	0.004	*F(1, 898) = 0.18*	0.67	0.000	[0.000, 0.006]	
Age group × Sex	3	0.006	*F(3, 898) = 0.30*	0.82	0.001	[0.000, 0.005]	
Family composition	4	0.123	*F(4, 898) = 6.24*	<0.001	0.027	[0.010, 0.047]	***
Location	4	0.017	*F(4, 898) = 0.87*	0.48	0.004	[0.000, 0.011]	
Home cooking frequency	1	0.116	*F(1, 898) = 5.89*	0.015	0.007	[0.001, 0.021]	*
Dining area	5	0.030	*F(5, 898) = 1.50*	0.19	0.008	[0.000, 0.018]	
Job type	4	0.033	*F(4, 898) = 1.67*	0.16	0.007	[0.000, 0.018]	
BMI	1	0.013	*F(1, 898) = 0.65*	0.42	0.001	[0.000, 0.008]	
Residual	898	0.020					

**Note.** Type III sums of squares. N = 925 (participants aged < 60 with complete data on all model variables). df = degrees of freedom; MS = mean square; η^2^p = partial eta-squared; CI = 95% confidence interval for η^2^p. Family size categories: 1, 2, 3, 4, ≥5 persons (df = 4). Job type categories: Administrative, Production/Technical, R&D, Sales, Other (df = 4). The Age group × Sex interaction term was non-significant (*p* = 0.822). * *p* < 0.05. *** *p* < 0.001.

**Table 3:** Association between family size and Shannon diversity index of food intake. Estimated marginal means of Shannon diversity index by family size and pairwise comparisons.

Family Size	EMM (Shannon)	95% CI (Lower–Upper)	F (df1 = 4, df2 = 900)/*p* for Family_Size	Partial η^2^ (Family_Size)	Significant Pairwise Differences (Tukey)
1 person	1.973	1.922–2.025	F = 6.45, *p* < 0.001	0.028	1 vs. 3, 1 vs. 4, 1 vs. 5+
2 persons	2.009	1.956–2.062			–
3 persons	2.03	1.976–2.084			1 vs. 3
4 persons	2.039	1.983–2.094			1 vs. 4
5+ persons	2.05	1.992–2.109			1 vs. 5+

Values are estimated marginal means (EMMs) of the Shannon diversity index adjusted for age group, sex, location, BMI, job type, homemade cooking, and dining area. *p* values for the main effect of family size are from a Type III ANOVA. Partial η^2^ indicates effect size.

**Table 4:** Dietary Intake Frequency by Household Type (single vs. multiple): Adjusted Comparisons Across Food Categories (ANCOVA).

**Food**	**Model 2**						
	**EMM** **(Single)**	**EMM** **(Multiple)**	**Diff**	**F**	**η^2^_p_**	***p* (FDR)**	**Sig.**
Meat	0.44	0.46	−0.023	1.44	0.0015	0.41	n.s.
Fish	0.18	0.20	−0.024	7.67	0.0078	0.013	*
Egg	0.26	0.29	−0.038	8.61	0.0087	0.010	*
Milk	0.71	0.68	0.022	0.56	0.0006	0.51	n.s.
Soy	0.54	0.58	−0.035	0.99	0.0010	0.41	n.s.
Vegetable	0.73	0.77	−0.037	0.99	0.0010	0.41	n.s.
Fruits	0.43	0.42	0.0093	0.11	0.0001	0.75	n.s.
Seaweed	0.38	0.45	−0.075	10.74	0.0109	0.005	**
Potato	0.32	0.39	−0.069	15.36	0.0155	<0.001	***
Oil	—	—	—	—	—	—	—
**Food**	**Model 3**						
	**EMM** **(Single)**	**EMM** **(Multiple)**	**Diff**	**F**	**η^2^_p_**	*p* **(FDR)**	**Sig.**
Meat	0.44	0.46	−0.023	1.427	0.002	0.38	n.s.
Fish	0.18	0.20	−0.024	7.621	0.008	0.013	*
Egg	0.26	0.2936	−0.037	8.235	0.008	0.013	*
Milk	0.70	0.68	0.021	0.489	0.0005	0.55	n.s.
Soy	0.54	0.58	−0.037	1.153	0.0012	0.38	n.s.
Vegetable	0.73	0.77	−0.039	1.102	0.0011	0.38	n.s.
Fruhits	0.42	0.42	0.0062	0.047	<0.001	0.83	n.s.
Seaweed	0.37	0.45	−0.077	11.17	0.0113	0.004	**
Potato	0.32	0.39	−0.070	15.77	0.016	<0.001	***
Oil	—	—	—	—	—	—	—

**Note.** EMM = estimated marginal mean; Diff = difference (EMM Multiple − EMM Single); η^2^p = partial eta-squared; *p* (FDR) = *p*-value adjusted by the Benjamini–Hochberg false discovery rate procedure. **Model 2** = adjusted for age and sex; **Model 3** = additionally adjusted for BMI. Oil was excluded from the analysis due to constant intake across groups. Highlighted rows indicate statistically significant group differences after FDR correction. *** *p* < 0.001; ** *p* < 0.01; * *p* < 0.05; n.s. = not significant.

**Table 5:** Interactive Effects of Household Type and Homemade Cooking on Dietary Diversity: Type III ANOVA.

Source	df	MS	F (df_1_, 903)	*p*	η^2^_p_	95% CI	Sig.
Intercept	1	42.39	—	<0.001	—	—	***
Single status	1	0.019	0.97	0.33	0.001	[0.000, 1.000]	n.s.
Homemade cooking	1	0.027	1.35	0.25	0.002	[0.000, 1.000]	n.s.
Age group	3	0.031	1.58	0.19	0.005	[0.000, 1.000]	n.s.
Sex	1	0.0093	0.47	0.49	<0.001	[0.000, 1.000]	n.s.
Location	4	0.020	1.04	0.39	0.005	[0.000, 1.000]	n.s.
Dining area	5	0.028	1.40	0.22	0.008	[0.000, 1.000]	n.s.
Job type	4	0.034	1.72	0.14	0.008	[0.000, 1.000]	n.s.
BMI	1	0.016	0.80	0.37	<0.001	[0.000, 1.000]	n.s.
Single status *×* Homemade cooking	1	0.084	4.28	0.04	0.005	[0.000, 1.000]	*
Residual	903	0.020	—	—	—	—	—

**Note.** Type III sums of squares. df = numerator degrees of freedom; denominator df = 903 for all effects. MS = mean square. F values are F(df_1_, 903). η^2^p = partial eta-squared with 95% bootstrap confidence interval. *p*-values are unadjusted. The shaded row indicates the significant interaction term. * *p* < 0.05; *** *p* < 0.001. n.s. = not significant; — = not applicable.

## Data Availability

Some or all datasets generated and/or analyzed during the current study are not publicly available due to their containing information that could compromise the privacy of research participants but are available from the corresponding author upon reasonable request.
